# New Finger Biometric Method Using Near Infrared Imaging

**DOI:** 10.3390/s110302319

**Published:** 2011-02-24

**Authors:** Eui Chul Lee, Hyunwoo Jung, Daeyeoul Kim

**Affiliations:** 1 Division of Fusion and Convergence of Mathematical Sciences, National Institute for Mathematical Sciences/463-1, Jeonmin-Dong, KT Daeduk Research Center, Yuseong-gu, Daejeon 305-390, Korea; E-Mail: daeyeoul@nims.re.kr; 2 Department of Math and Computer Science, Korea Science Academy of KAIST, Pusan, Korea; E-Mail: hwjung@nims.re.kr

**Keywords:** finger recognition, finger vein, finger geometry, modified Gaussian high-pass filter, binarization, local binary pattern, local derivative pattern

## Abstract

In this paper, we propose a new finger biometric method. Infrared finger images are first captured, and then feature extraction is performed using a modified Gaussian high-pass filter through binarization, local binary pattern (LBP), and local derivative pattern (LDP) methods. Infrared finger images include the multimodal features of finger veins and finger geometries. Instead of extracting each feature using different methods, the modified Gaussian high-pass filter is fully convolved. Therefore, the extracted binary patterns of finger images include the multimodal features of veins and finger geometries. Experimental results show that the proposed method has an error rate of 0.13%.

## 1. Introduction

To guarantee a highly secure authorization and identification system, biometrics has been used in many kinds of applications such as door lock systems, financial activities, and immigration control. Biometrics is a type of identification or verification method that uses human physiological and behavioral features such as fingerprints, faces, irises, gaits, and veins [1].

Biometric methods can be divided into two main categories: physiological and behavioral methods. Physiological methods are related to the shape of the human body. The characteristics focused upon in these methods include fingerprints, faces, DNA, hand and palm geometries, and irises. Behavioral methods are related to human behavior, including typing rhythm, gait, and voice characteristics. Behavioral characteristics are also called “behaviometrics” [2]. Strictly speaking, the human voice is also a physiological characteristic because every person has a different vocal tract; however, voice recognition is mainly based on the study of the way people speak, and it is commonly classified as a behavioral characteristic [3].

Such biometric methods have both advantages and disadvantages. Although iris recognition has a high recognition accuracy, iris-capturing devices are relatively expensive. A fake iris imitating a pre-captured iris image, such as a printed fake iris, patterned contact lens, or artificial eye, can cause an iris recognition system to generate a false alarm. Furthermore, certain occlusion factors such as eyelashes [4], specular reflections [5], and eyelids [6] can degrade the performance of iris recognition systems.

Fingerprint recognition also has high recognition accuracy, and such sensing devices are relatively cheap. However, moisture or scars can prevent a clear fingerprint pattern from being obtained [7]. Moreover, fingerprints can also be used for criminal or other illegal purposes since fingerprint patterns can be easily obtained from certain surfaces [8]. Recently, new finger biometric methods using finger-knuckle-prints have been introduced [9,10]. In these researches, the authors used finger-knuckle-prints, which are the skin patterns of the outer surface area of a phalangeal joint. They achieved promising recognition accuracy using Gabor-based feature extraction.

Face recognition is comparatively convenient in terms of usability; however, the recognition accuracy of common face recognition systems is even lower than that of other systems such as iris or fingerprint recognition methods. Moreover, since faces are always exposed, it is easy to trick a recognition system [11] using a photograph or mask. Several factors such as facial expressions [12], illuminative variations [13], aging effects [14], and occlusions from wearing masks or glasses [15] can degrade the performance of face recognition systems.

The reported accuracies of other behavioral methods such as voice [16], gait [17], and keystroke recognition [18] have been quite poor. The current health status of the subject, along with certain environmental conditions, can significantly degrade the performance of these recognition systems, rendering their use difficult.

To overcome the problems of previous biometric systems, new systems using vein patterns from the palms or hands have been introduced [19] and are continuously being researched [1,20–24]. In general, vein patterns can be acquired using near-infrared (NIR) light and a camera device. However, the size of the device should be sufficiently large, as palm and hand vein recognition methods require the users to place their hand on the device in order to capture the entire hand region.

To overcome the problems of vein recognition systems, finger vein recognition methods have been researched [1,25]. Yanagawa *et al*. proved that each finger from the same person has unique vein patterns [26]. Miura *et al*. proposed a finger vein extraction method using repeated line tracking [27]. Zhang *et al*. proposed a finger vein extracting method based on curvelet information of the image profile and locally interconnected a structural neural network [28]. Recently, Miura reported that finger vein thickness could be altered by blood flow or weather conditions [29]. He also proposed a finger vein pattern extraction method that allows for various pattern thicknesses [29]. In addition, a commercial product was introduced by Hitachi [30,31]. In our previous research, a local binary pattern (LBP)-based finger vein recognition method was proposed, in which a binary pattern was extracted from a stretched rectangular finger region [24]. Further, a modified Hausdorf distance (MHD)-based minutiae matching method has been used, in which vein pattern extraction should be performed to extract minutiae (bifurcation and ending) points [25]. According to previous finger vein recognition methods, vein-pattern or finger-region extraction procedures should be performed for feature extraction or matching. Vein pattern extraction procedures increase the time complexity. Moreover, if a finger image includes noise factors such as shadows or fingerprints, a falsely extracted pattern may occur, degrading the recognition accuracy. Even in finger-region extraction methods, stretched quadrangle finger vein images include distortions due to the stretching procedure [1].

Therefore, in a previous research [1], features of finger veins, finger geometry, and fingerprints were extracted using a Gabor filter, and the hamming distance based on binarization was used for matching. However, since the directions and widths of finger veins vary, it is difficult to determine the optimal directions and frequencies of the Gabor filter. Further, the extracted binary codes of the same finger region obtained through binarization can be altered owing to local shadows on the finger area [1].

**Figure 1.**
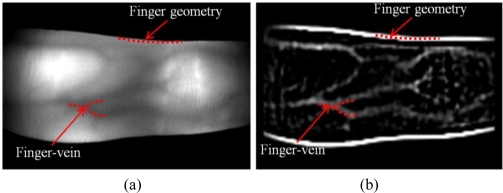
Example of finger geometry and finger veins components in **(a)** a captured IR finger image and **(b)** an image after modified Gaussian high-pass filtering.

To solve these problems, we propose a new finger recognition method. In addition to vein patterns, IR finger images also have features reflecting section of geometrical finger edge information, as shown in Figure 1. Among these three components, finger geometry appears most clearly. Furthermore, finger vein patterns are totally less clearly appeared. Because these two components include a brightness change factor, their features can be extracted using a single high-pass filter. Consequently, instead of performing a separate localization procedure for each component, an appearance-based method is selected. Therefore, we say that the proposed method is regarded as finger recognition and not for finger vein recognition. Instead of extracting each feature of a finger vein and finger geometry using different methods, a modified Gaussian high-pass filter is used. To represent the features in binary code, simple binarization, LBP and LDP methods are compared on the basis of the hamming distance (HD).

As shown in Figure 1(a,b), since parts of the finger geometry and finger vein are high frequency components, their modified Gaussian high-pass filtering results contain high values. Therefore, to extract a finger pattern using LBP, LDP, or binarization, pixels from not only certain sections of the finger but also the entire filtered image are used. That is, all high-pass filtered values around the finger edge and finger vein are reflected in generating separable binary finger patterns.

## 2. Proposed Method

### 2.1. IR Finger Imaging

**Figure 2.**
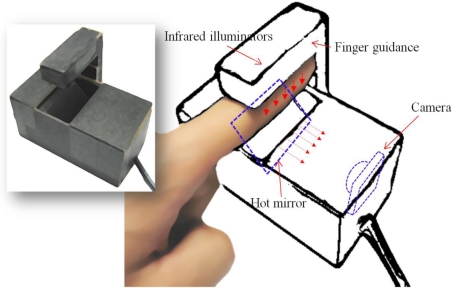
Finger imaging device.

We designed an IR finger vein imaging device in our previous works, which includes IR illuminators, a suitable camera with an IR pass filter, and a hot-mirror, as shown in Figure 2.

The IR illuminators are located on the finger dorsum, and IR light penetrates the finger. Both reflected and penetrating light are captured by a camera. In our system, the finger position within the captured image is important; there are no additional image alignment procedures. Therefore, our device has a finger dorsum and fingertip guide, and alignment of the finger images is guaranteed.

As shown in Figure 2, a hot mirror is positioned at 45° in front of the camera. The hot mirror was adopted to reduce the height of the capturing device. The mirror reflects IR light while allowing visible light to pass through. Using the hot mirror and illuminator module shown in Figure 2, the size of the recognition system could be greatly reduced.

The proposed device has a charge-coupled device (CCD) web camera, which adopts a universal serial bus (USB) interface, and since finger vein patterns are visible using NIR light, a original visible light passing (NIR rejection) filter of the web camera is removed. Instead, an NIR passing filter (Visible light rejection) is included inside the camera, which allows only NIR light with wavelengths greater than 750 nm to pass. To make the finger vein patterns more distinctive, five additional NIR light-emitting diodes (LEDs) are attached to the upper part of the device, as shown in Figure 1. Considering a trade-off between image brightness due to the sensing ability of the CCD sensor and the absorption amount of deoxygenated hemoglobin [32], we chose NIR illuminators with 850 nm wavelengths.

Our system down-samples captured images from 640 × 480 pixels to 128 × 96 pixels in order to reduce the time complexity and eliminate pixel noise [1].

**Figure 3.**
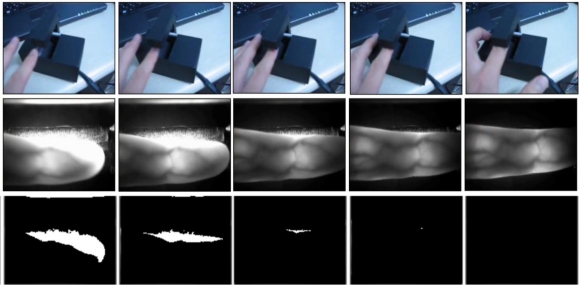
Procedure used for acquiring an adequate finger image from successive images. The first row shows successive images of a user placing his finger on the device. The second row shows the corresponding finger images. The third row shows the results of binarization using a threshold of 250.

To acquire adequate finger images, we reduce the finger alignment problem and the amount of saturated illumination by performing binarization for every input image using a threshold of 250. Since a filter allowing NIR light to pass through is attached to the camera, and the gray levels of the highly saturated regions are higher than those of the other areas, the binarization procedure can estimate the amount of saturation. The last image in Figure 3 is finally accepted for further processing because it does not include a saturated area.

### 2.2. Finger Image Enhancement

**Figure 4.**
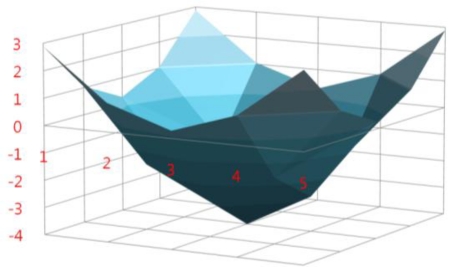
Visualization of modified 5 × 5 pixel Gaussian high-pass filter (*a* = 10.9, *b* = −4).

A modified Gaussian high-pass filter is used to enhance features such as finger geometry and finger veins. In a previous work [1], the authors used a Gabor filter for image enhancement. However, since the directions and widths of finger veins vary, it is difficult to determine the optimal directions and frequencies of a Gabor filter. Because Gabor filters are geometrically composed using a combination of sinusoids with varying frequencies, they are conventionally used to extract not only mid-frequency features but also very high frequency components such as the fine wrinkles present on a palm print [33] and iris muscle patterns for iris recognition [34]. In comparison, since the proposed modified Gaussian high-pass filter doesn’t have all combinations of the various sinusoids, as shown in Figure 4, this filter has an advantage in terms of extracting comparatively lower-frequency components such as finger edges or finger veins robust to high-frequency noise components.

To overcome these problems, we use a symmetrical modified Gaussian high-pass filter for image enhancement. The filter has the following formula:

(1) $$H(x,y)=a(1-e^{-D^{2}(x,y)/2D_{0}^{2}})+b$$

Here, *D*(*x*, *y*) is defined using the following equation:

(2) $$D(x,y)={\left[{\left(x-x_{0}\right)}^{2}+{\left(y-y_{0}\right)}^{2}\right]}^{1/2}$$

where *x* and *y* are the positions relative to the center ((*x_0_*, *y_0_*)) of a convolution mask, *D*(*x*, *y*) represents the distance between the center and a relative position, and *a* and *b* are adjustment variables that can change the amplitude and DC level of the filtering mask. In a mask of size 5 × 5 pixels, the optimal values of *a* and *b* are empirically defined as 10.9 and −4, respectively. To determine *a* and *b* values, we firstly consider that the summation of coefficients of the filter should be 0. If the summation is greater than 0, the average brightness of filtering result image may be increased. In contrast, the summation having lower than 0 may make comparatively dark result. The values of 10.9 and −4 satisfy this condition of the filter. However, there can be exist the other combination of *a* and *b* values which satisfy the condition. Therefore, we secondly consider the visibility of finger geometry and finger vein in the filtered image. Through our some test, the values of 10.9 and −4 showed the best visibility of finger geometry and finger vein. The corresponding modified Gaussian high-pass filtering mask is visualized in Figure 4.

**Figure 5.**
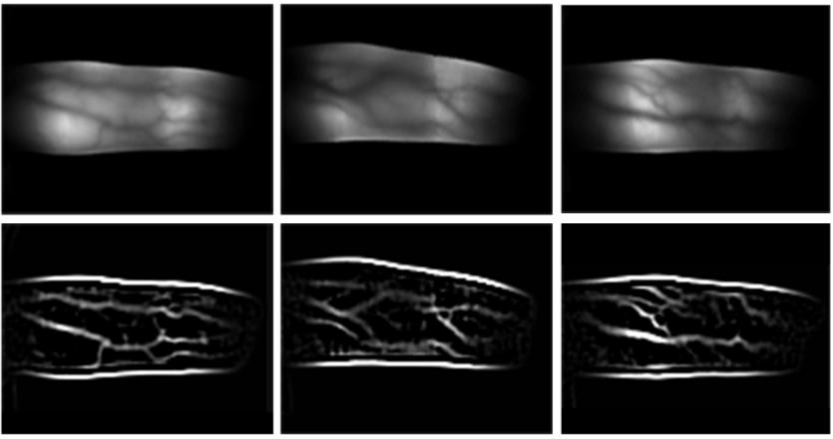
Down-sampled finger images (top) and their filtered results.

**Figure 6.**
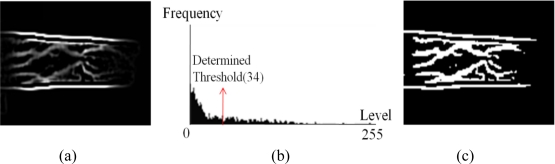
Example of simple binarization method. **(a)** Filtered finger image, **(b)** histogram of image in (a) and an automatically determined threshold, and **(c)** a resulting image after binarization using the threshold.

The designed 5 × 5 pixel filtering mask fully convolves IR finger images of size 128 × 96 pixels. Examples of finger images and their convolved results are shown in Figure 5. There are small amounts of noise components in the three fingers’ images in Figure 5. These are caused by undesirable reflections from IR illumination on the outer surfaces of the fingers. However, since their calculated amplitudes after filtering are smaller than those in the finger regions, as shown in Figure 5, these noise components can be easily removed through the binarization process shown in Figure 6.

### 2.3. Binary Feature Extraction

Since a filtered finger image still has 256 levels, we perform a binary code extraction procedure after filtering. In the previous work [1], when binarization is used, the extracted binary codes of the same finger region achieved through binarization can be changed owing to local shadows appearing on the finger area. To overcome this problem, we compared the performances of simple binarization, LBP method, and LDP method.

In the first method, *i.e*., simple binarization, the robust threshold value is important. Gonzalez and Wood proposed a method for automatically determining the threshold for cases in which the foreground and background of an image are clearly separated [1,35]. The threshold (*T*) determination method for binarization is operated as indicated in the following procedure [35]:

1. Select an initial estimate for the global threshold, *T*.
2. Segment the image using *T*. This will produce two groups of pixels: *G_1_* consisting of all pixels with intensity values > *T*, and *G_2_* consisting of pixels with values ≤ *T*.
3. Compute the average (mean) intensity values *m1* and *m2* for the pixesl in *G_1_* and *G_2_*, respectively.
4. Compute a new threshold value by *T* = (*m_1_* + *m_2_*)/2
5. Restep 2 through 4 until the difference between value of *T* in successive iterations is smaller than a predefined parameter *ΔT*.

Figure 6 shows a filtered image and a corresponding image that was binarized using the thresholding method. Consequently, binary finger codes of 12,288 (128 × 96) bits are generated from finger images of size 128 × 96 pixels.

**Figure 7.**
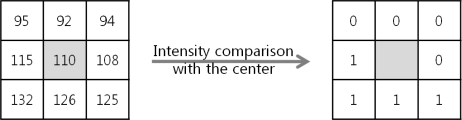
Example of an LBP operator.

Next, we considered the LBP extraction method. Ojala *et al*. proposed an LBP operator as a nonparametric 3 × 3 kernel for texture classification [36,37]. An LBP can be defined as an ordered set of binary values determined by comparing the gray values of a center pixel and the eight neighboring pixels around the center, as shown in Figure 7. An ordered set of binary values can be expressed in decimal form as shown in Equation (3) [23]:

(3) $$\mathrm{LBP}(x_{c},y_{c})=\sum_{n=0}^{7}s(i_{n}-i_{c})2^{n}$$

where *i_c_* and *i_n_* denote the gray value of the center pixel (*x_c_*, *y_c_*) and those of the eight neighboring pixels, respectively. The function *s*(*x*) is defined as [23]:

(4) $$s(x)=\{\begin{array}{lll} 1 & \mathrm{if} & x\geq 0\\ 0 & \mathrm{if} & x<0 \end{array}$$

Through Equations (3) and (4), the LBP extracts a finger binary code of 94,752 bits. These 94,752 bits are calculated as 126 (the number of kernel movements in the X direction) × 94 (the number of kernel movements in the Y direction) × 8 (the number of calculated bits from one position of kernel) in an image of size 128 × 96 pixels. In Figure 7, the binary sequence on the 3 × 3 block is defined clockwise from the top-left as 00001111_(2)_.

**Figure 8.**
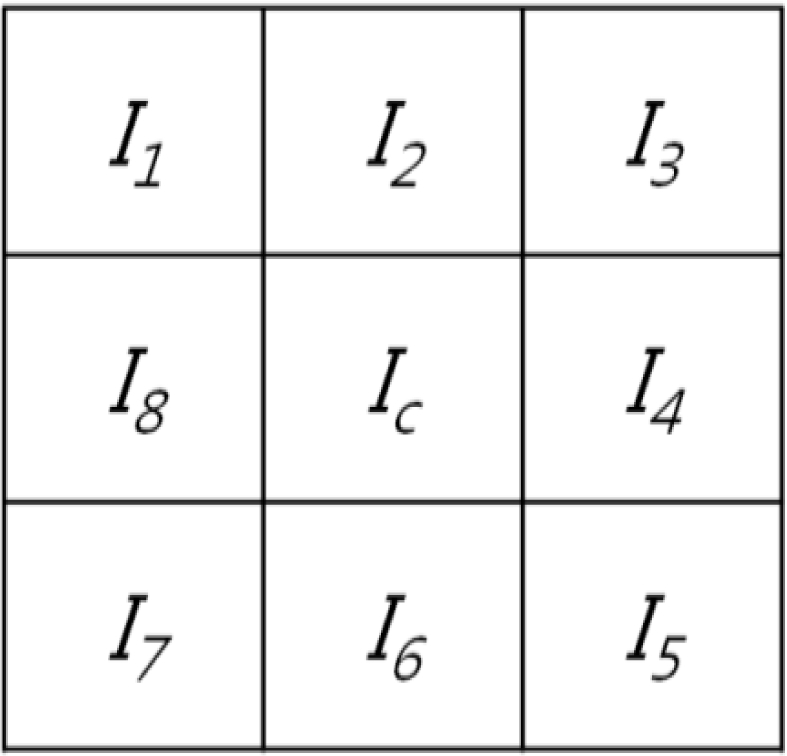
Eight adjacent pixels around *I_c_*.

Next, the LDP extraction method is adopted [38,39]. The LDP represents a high-order derivative pattern occurring in a specific direction, which is reported to extract more elaborate and discriminative features than those by the LBP [39]. In this study, the codes are extracted from a filtered image using a second-order LDP, considering the 0°, 45°, 90°, and 135° directions. If the eight adjacent pixels are positioned around the center position (*I_c_*), as shown in Figure 8, the first-order derivative bits along each direction are defined as [39]:

(5) $$B_{0{}^{\circ}}\;\left(x_{c},y_{c}\right)=f\left(I_{4}-I_{c}\right)$$

(6) $$B_{45{}^{\circ}}\;\left(x_{c},y_{c}\right)=f\left(I_{3}-I_{c}\right)$$

(7) $$B_{90{}^{\circ}}\;\left(x_{c},y_{c}\right)=f\left(I_{2}-I_{c}\right)$$

(8) $$B_{135{}^{\circ}}\;\left(x_{c},y_{c}\right)=f\left(I_{1}-I_{c}\right)$$

(9) $$f\left(k\right)=\{\begin{array}{cc} 1, & k>\mathrm{th}\\ 0, & k\leq \mathrm{th} \end{array}$$

where (*x_c_*, *y_c_*) and *th* denote the position of the center pixel *I_c_* and a predefined threshold, respectively. The predefined threshold was set to 0 in our experiment. The LDP extracts the feature codes from an exclusive-OR (⊗) operation of the corresponding first-order derivative bits between the center pixel and eight adjacent pixels.

Based on the above method, LDP features can be generated using the following equations [39]:

(10) $${\mathrm{LDP}}_{\alpha }\;\left(x_{c},y_{c}\right)=\sum_{i=1}^{8}\left\{B_{\alpha }\;\left(x_{c},y_{c}\right)⊗B_{\alpha }\;\left(x_{c}+u_{i},\;y_{c}+v_{i}\right)\right\}\cdot 2^{i-1}$$

(11) $$u_{a}=\{\begin{array}{cc} -1, & \mathrm{if}\;a=1\\ 0, & \mathrm{if}\;a=2\\ 1, & \mathrm{if}\;a=3\\ 1, & \mathrm{if}\;a=4\\ 1, & \mathrm{if}\;a=5\\ 0, & \mathrm{if}\;a=6\prime \\ -1, & \mathrm{if}\;a=7\\ -1, & \mathrm{if}\;a=8 \end{array}v_{a}=\{\begin{array}{cc} -1, & \mathrm{if}\;a=1\\ -1, & \mathrm{if}\;a=2\\ -1, & \mathrm{if}\;a=3\\ 0, & \mathrm{if}\;a=4\\ 1, & \mathrm{if}\;a=5\\ 1, & \mathrm{if}\;a=6\\ 1, & \mathrm{if}\;a=7\\ 0, & \mathrm{if}\;a=8 \end{array}$$

(12) $${\mathrm{LDP}}_{\alpha }\;\left(x_{c},y_{c}\right)=\left\{{\mathrm{LDP}}_{\alpha }\;\left(x_{c},y_{c}\right)|\alpha =0^{{}^{\circ}},\;45^{{}^{\circ}},\;90^{{}^{\circ}},\;135^{{}^{\circ}}\right\}$$

Consequently, the LDP extracts a finger binary code of 365,056 bits. These 365,056 bits are calculated as 124 (the number of kernel movements in the X direction) × 92 (the number of kernel movements in the Y direction) × 4 (considered directions) × 8 (extracted bits per direction) in an image of size 128 × 96 pixels.

### 2.4. Matching

The proposed method measures the HD in order to estimate the similarities between the extracted binary codes and the enrolled code [1]. The HD is generally adopted to measure dissimilarities between two binary patterns [34], which are represented using the following formula:

(13) $$\mathrm{HD}=\frac{\left\|\left(\mathrm{codeA}⊗\mathrm{codeB}\right)\right\|}{\mathrm{CodeLength}}$$

In Equation (13), ⊗ is a Boolean exclusive-OR operator between two binary patterns. Therefore, HD can be calculated by dividing the number of binary codes (*CodeLength* = Binarization: 12,288; LBP: 94,752; LDP: 365,056). Therefore, the HD ranges from 0 to 1.

Although we adopted a procedure for acquiring adequate finger images, as shown in Figure 3, finger-image alignment may not be perfectively achieved. In contrast to [1], these misalignment problems are solved through bit shifting in the matching stage using the HD. Among the HD values calculated through bit shifting, the minimum value is chosen as the final HD.

## 3. Experimental Results

**Figure 9.**
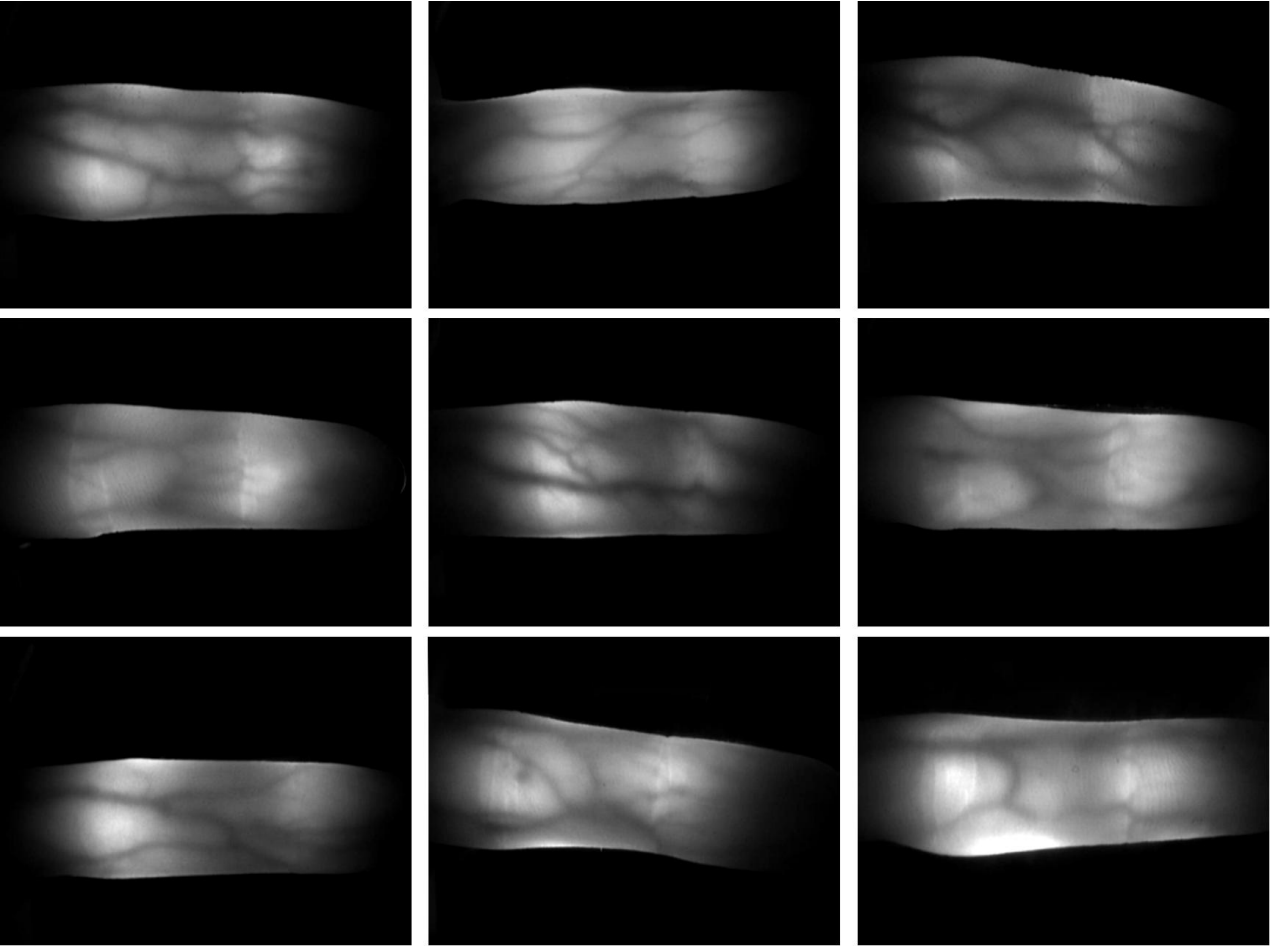
Examples of IR finger images.

To test the proposed finger recognition method, we collected 10 images from 30 subjects. Since the length of the thumb is too short to be captured using our device, the two thumbs of each subject were not collected. To collect natural finger images in terms of variations in position and illumination, the images were captured over a certain time interval. Consequently, the collected database includes 2,400 finger images (240 classes (30 persons × 8 fingers) × 10 images). The spatial and depth resolutions of the captured finger images were 640 × 480 pixels and 256 gray levels, respectively [1]. The test program was operated using an Intel Core i7 (2.67 GHz) CPU with a 6 GB RAM. Figure 9 shows examples of finger images captured using our device.

In the first test, the total processing time was measured as shown in Table 1. The processing time of the three feature extraction schemes, simple binarization, LBP, and LDP, was compared. The total processing time of simple binarization was 30.6 ms; this was the best result, *i.e*., fastest processing, among the three methods. The processing time of the LBP-based method was 44.7 ms, whereas that of the LDP-based method was 112.5 ms. These results were foreseeable as LBP and LDP extract a larger number of codes than the binarization method.

**Figure 10.**
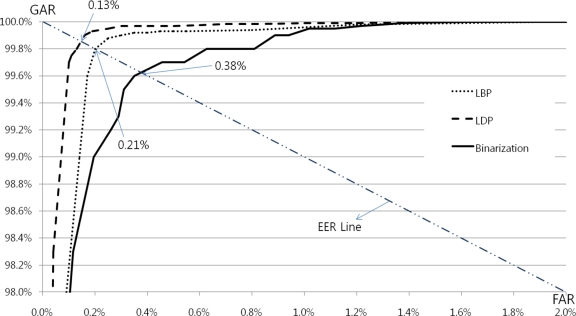
ROC curves of experimental results using simple binarization, LBP, and LDP.

Next, a recognition test was performed. We measured the recognition accuracy in terms of the receiver operating characteristic (ROC). As shown in Figure 10, the ROC includes the genuine acceptance rate (GAR) and a false acceptance rate (FAR) [40]. The GAR is defined as 100%—false-rejection rate (FRR). An FAR indicates an error occurring when an un-enrolled finger image is accepted as an enrolled image. The FRR indicates the number of errors that occur when enrolled finger images are rejected as un-enrolled images.

As shown in Figure 10, the EERs achieved using the LDP, LBP, and binarization methods were 0.13%, 0.21%, and 0.38%, respectively. The LBP- and LDP-based pattern extraction methods were better than the simple binarization method as they also considered neighboring pixels. Furthermore, the LDP analysis was more reliable than LBP because the LDP method also considered the directions of the adjacent pixels. All results were yielded from the same database and experimental protocols. In our test, genuine tests were performed 10,800 (240 classes × _10_C_2_) times, whereas imposter tests were performed 2,868,000 (_2,400_C_2_ − 10,800) times.

**Figure 11.**
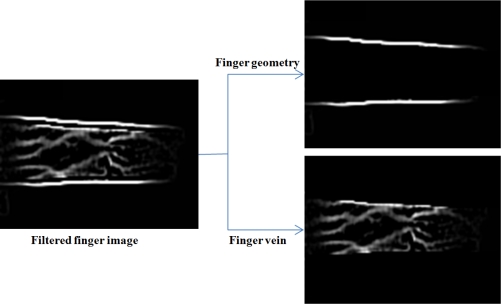
An example of manually separating filtered finger image into two component images such as finger geometry and finger vein.

Next, we performed the additional experiment in order to verify the performance of two features (finger vein, and finger geometry). To separate two features, we manually divided each filtered finger image into two component images as shown in Figure 11.

Then according to recognition method and used component, we acquired EERs to verify the contribution of finger geometry component as shown in Table 2.

Based on the results of Table 2, our proposed method using both finger geometry and finger vein showed the best result compared with the other two cases of using only finger geometry or finger vein. Even though the case of using only finger geometry showed very low recognition accuracies, the proposed method using both components showed the increased performance than the cases of using only finger vein. Consequently, we found that the finger geometry made a positive contribution in terms of recognition accuracy.

## 4. Conclusions

In this study, a new finger recognition method based on a modified Gaussian high-pass filter was developed. After the filtering procedure, three types of feature extraction methods—simple binarization, LBP, and LDP—were adopted and compared. Logically, an extracted binary pattern includes multimodal biometric features of finger veins and finger geometries.

Our method has the following novelties compared with previous research results: firstly, our method shows improved recognition accuracy compared with only using finger vein patterns by logically including both finger vein and finger geometric components. Secondly, the processing time was reduced by using whole finger images without localizing any region of interest. Thirdly, the proposed modified Gaussian high pass filter strongly enhanced finger vein and finger geometry components which considered whole directions compared with the conventionally used directive Gabor filter. Fourthly, our method showed promising recognition performance without any finger alignment algorithm by using adequate finger image capturing scheme.

Experimental results showed that the LDP-based method was the best in terms of recognition accuracy, whereas the simple binarization scheme was the best in terms of processing time. In future works, we intend to consider a software-based alignment method based on the affine transform model using binary patterns.

## Figures and Tables

**Table 1.** Processing times.

**Simple binarization**	**LBP**	**LDP**
30.6 ms	44.7 ms	112.5 ms

**Table 2.** EERs according to kinds of component and recognition method.

**Recognition method**	**Binarization**	**LBP**	**LDP**
**Used component**			
Finger geometry	23.16%	22.98%	17.86%
Finger vein	2.32%	1.53%	0.89%
Finger geometry + Finger vein	0.38%	0.21%	0.13%
